# Lactoferrin—A Regulator of Iron Homeostasis and Its Implications in Cancer

**DOI:** 10.3390/molecules30071507

**Published:** 2025-03-28

**Authors:** Izabela Bolesławska, Natasza Bolesławska-Król, Karol Jakubowski, Juliusz Przysławski, Sławomira Drzymała-Czyż

**Affiliations:** 1 Department of Bromatology, Poznan University of Medical Sciences, 60-806 Poznan, Poland; jakubowski.karol@ump.edu.pl (K.J.); jprzysla@ump.edu.pl (J.P.); drzymala@ump.edu.pl (S.D.-C.); 2 Student Society of Radiotherapy, Collegium Medicum, University of Zielona Góra, Zyta 28, 65-046 Zielona Góra, Poland; natmeff@gmail.com

**Keywords:** iron, lactoferrin, anticancer, antioxidant, anti-inflammatory effects, effects on the microbiome, anemia resulting from cancer

## Abstract

Cancer is a global health challenge, and its development is closely linked to iron metabolism. Cancer cells have an increased demand for this element, which promotes their proliferation, invasion, and metastasis. Excess iron catalyzes the formation of reactive oxygen species (ROS), which can both induce ferroptosis and initiate oncogenic signaling pathways. The deregulation of iron metabolism in cancer patients leads to anemia or toxic iron overload and also affects the gut microbiota. Lactoferrin (LF), a glycoprotein with strong iron chelating properties, can regulate its availability to cancer cells, thereby limiting their growth and progression. By chelating free Fe ions, LF reduces oxidative stress and inhibits the mechanisms that promote carcinogenesis. Additionally, it exhibits immunomodulatory and anti-inflammatory effects and may enhance the body’s anti-tumor response. This review analyses the mechanisms of action of lactoferrin in the context of cancer, with a particular focus on its chelating, antioxidant, and immunomodulatory properties. The multidirectional effects of LF make it a promising component of preventive and therapeutic strategies, requiring further clinical studies.

## 1. Introduction

Estimates of the global burden of cancer published by the World Health Organization (WHO) and the International Agency for Research on Cancer (IARC) show a steady increase in the number of cancer cases. In 2022, there were 20 million new cases and 9.7 million cancer deaths. The estimated number of people with five-year survival after diagnosis was 53.5 million [1]. These data indicate an urgent need for increased research into effective cancer prevention and treatment strategies.

Cancer biology and iron are interrelated through multiple pathways. While iron (Fe) is an essential element for the normal course of key biological processes [2,3,4], a hallmark of cancer is altered systemic iron metabolism [5,6]. Cancer cells are characterized by an increased requirement for iron, which is essential for their proliferation, invasion, and metastatic processes [7]. Accordingly, tumors develop advanced mechanisms of iron recruitment, transport and storage [8], resulting in a disruption of homeostasis of this element in the host, manifesting as iron deficiency or excess [9]. In addition, the high metabolism and proliferation rate of tumor cells activating various mechanisms to obtain the iron required for tumor growth catalyzes the formation of reactive oxygen species (ROS) through Fenton/Haber–Weiss chemistry [10], which can result in both iron-induced cell death (ferroptosis) and the initiation of oncogenic signalling pathways, leading to cellular damage, inflammation, and cancer progression [11,12,13]. In addition, the recognition of tumor cells and neoantigens by the immune system results in the production of numerous inflammatory cytokines [14,15]. These cytokines contribute to specific perturbations of iron homeostasis, affecting iron flow in the mononuclear phagocytic system. The result can be the development of hypoferremia (reduction in circulating iron levels) and hyperferritinemia (excessive accumulation of iron in the body) [11,12,13]. Iron abnormalities also affect the gut microbiota—both excess and deficiency can lead to dysbiosis [16].

One common complication in oncology patients is anemia due to reduced iron availability or functionality, particularly among those undergoing chemotherapy [17]. In turn, iron overload can lead to additional complications and an increased risk of death [9]. The dual—both pro- and anti-cancer—role of iron, combined with multidirectional regulatory mechanisms, leads to unpredictable interactions in the course of cancer. Furthermore, previous therapeutic strategies targeting the stabilization of iron homeostasis and the modulation of cellular redox status have not yielded satisfactory results, highlighting the need to search for new therapeutic approaches.

A promising direction for research into cancer prevention and therapy is the use of food components with documented anticancer properties. One such compound is lactoferrin (LF), a glycoprotein present in body fluids and mucosal secretions of mammals and characterized by strong iron-chelating properties. Numerous studies have demonstrated its immunomodulatory and anti-inflammatory [18,19], anticoagulant [20], hypotensive [21], and antidiabetic [22] effects. In addition, LF exhibits bacteriostatic, bactericidal, and antiviral activity, including against the SARS-CoV-2 virus [23,24].

LF crosses from the blood into the cerebrospinal fluid through the blood–brain barrier [25], where its beneficial effects have been documented in the context of neurodegenerative diseases such as Parkinson’s disease, Alzheimer’s disease, and dementia, as well as in depression and multiple sclerosis [26]. It has also been shown to have anticancer effects [27,28].

This review focuses on recent reports of the anticancer properties of lactoferrin resulting from its effects on iron metabolism. Particular attention has been given to its chelating, anti-inflammatory, and antioxidant effects, as well as its impact on the gut microbiota. In addition, the role of lactoferrin in inhibiting cancer-associated anemia was analyzed and the available evidence on its efficacy and safety was assessed in the context of potential applications.

## 2. Materials and Methods

In this narrative review, we collected available peer-reviewed articles that addressed the antitumor effect of lactoferrin through its effect on iron. Articles were collected from recommended databases including PubMed, MEDLINE, Elsevier, Google Scholar, Science Direct, MDPI databases, Scopus, and Web of Science. The main keywords used in this review were: lactoferrin, iron, anti-cancer, antioxidant, anti-inflammatory, and effect on the microbiome.

We conducted a narrative review of the role of lactoferrin as a regulator of iron homeostasis in cancer therapy covering publications from 2000 to the present. The same time frame covered a review of selected studies on the antioxidant properties of lactoferrin in in vitro and human models, as well as its efficacy and safety in the treatment of anemia. Due to the cumulative nature of the reviews, priority was given to the most recent work from the last five years. Older references (before 2000) were also included, but only on the basis of their relevance, with particular emphasis on primary publications.

The inclusion criteria included original research articles in English, available in full text, focusing on the role of lactoferrin as a regulator of iron homeostasis in cancer therapy.

Exclusion criteria included publications in languages other than English, conference abstracts, editorials, non-peer-reviewed papers, duplicates, and, in the case of reviews of studies on the properties of lactoferrin in in vitro and human models, as well as its effectiveness in the treatment of anemia, those not presenting quantitative data.

## 3. Iron in the Human Body

Iron is an essential element for the normal functioning of the human body, occurring in the body under physiological conditions in an amount of 3–4 g [29]. It is a component of hemoproteins and iron-sulfur proteins responsible for many functions, ranging from oxygen transport to cell metabolism and DNA synthesis; it is included in the prosthetic groups of the active centers of enzymes and non-heme compounds [2,3,4]. Under physiological conditions, approximately 70%–80% of the body’s iron is used for the production of hemoglobin, the main component of erythrocytes, and it is also found in myoglobin and iron-containing enzymes. Approximately 20% of the iron is a stock pool of iron located as ferritin and hemosiderin in the cytoplasm of Kupffer cells in the liver, spleen macrophages, and bone marrow [30,31]. A small amount of iron, less than 0.1%, is found in plasma, mostly bound to transferrin (TF), a protein that transports iron to the liver, bone marrow, and other tissues [32]. Approximately 2% of the iron in the body is found in the so-called labile iron pool, comprising free plasma iron unbound to transferrin (NTBI) and other traditional iron-binding proteins [33]. In addition, it is found in a non-permanent pool of redox-active iron complexes (LIPs), comprising both Fe^2+^ and Fe^3+^ ions bound to a variety of ligands [34]. Both of these intractable forms of iron can participate in the Fenton reaction, leading to the formation of reactive oxygen species [31].

Both excessively low and excessively high levels of systemic iron show a positive correlation with various diseases. Iron deficiency resulting from low dietary intake, chronic blood loss, malabsorption, or from increased demand can cause iron-restricted erythropoiesis leading to anemia [32], weakening the immune system and reducing the effectiveness of treatment [35]. Conversely, excess iron in the body leads to systemic iron overload and organ damage [36], and in the case of cancer, this can promote tumor growth and create an environment that protects cancer cells from treatment [35]. Therefore, maintaining blood iron levels at an appropriate level is crucial for normal body homeostasis. To protect itself from fluctuations in iron levels, the human body has developed a number of complex mechanisms that tightly control iron absorption, distribution, and storage, but it does not have an active iron removal system. Only a small part of it is excreted in urine, exfoliating epidermis and intestinal epithelium, and during bleeding.

Most of the iron utilized by the body (20–25 mg/day) is recovered from phagocytosed and degraded erythrocytes by splenic and hepatic macrophages [37,38]. In addition, about 10–15 mg of iron is supplied from the diet, of which only 10%, or about 1–2 mg of iron, is absorbed in the duodenum and proximal part of the jejunum [6,32].

The iron supplied by food, depending on its origin (plant/animal), can be in the form of heme iron or non-heme iron [39,40]. Non-heme iron occurring mostly in the form of Fe^3+^ has to be reduced to Fe^2+^ with the involvement of duodenal cytochrome b reductase 1 (DCYTB), after which it can only be absorbed in the more alkaline environment of the small intestine, mainly in enterocytes [41], and further enter the body via the divalent metal transporter 1 (DMT1) [42].

Heme iron has a higher bioavailability than non-heme iron, reaching 15–35% [39]. In contrast to the defined uptake pathway of non-heme iron, the absorption of heme iron is unclear. It is assumed to enter cells by receptor-dependent endocytosis and membrane transport [43]. Heme is then degraded by heme oxygenase 1 (HO-1), resulting in the release of Fe^2+^, carbon monoxide, and biliverdin [44]. Furthermore, ferrous iron is metabolized via the same pathway as non-heme iron [45].

### 3.1. Systemic Iron Turnover

After absorption by intestinal enterocytes, iron can be stored within ferritin (FT), a protein that functions as an iron store in hepatocytes. Alternatively, it can be immediately utilized in intracellular metabolic processes or released into the systemic circulation through the basolateral membrane of enterocytes with the involvement of ferroportin (FPN1), which is the only known exporter of iron from the cell interior into the bloodstream [46]. The FT structure comprises L and H subunits, which combine in different proportions to form a cage-like complex. This structure allows Fe^3+^ ions to bind in their inert form, which reduces the formation of reactive oxygen species and minimizes potential oxidative damage [38,46]. The amount of serum ferritin is negligible but proportional to intracellular storage and can thus be a marker of iron deficiency/excess used in clinical practice to assess iron metabolism [47]. Ferroportin binds and stores excess iron (approximately 10 per cent of total body iron) until there is a need to use it. It is found in both intracellular and extracellular compartments [48]. It can store up to 4500 iron atoms.

Basolateral iron transport is regulated by hepcidin, a peptide hormone dependent on the level of iron stored in the body. The high iron content in hepatocytes and its large body reserves result in increased synthesis of hepcidin, which, by binding FPN1, limits the release of iron into the circulation [6]. This action prevents toxicity associated with excessive iron levels and reduces its availability to pathogens [49,50], but at the same time, it can lead to iron-limited erythropoiesis and anemia [51]. Hepcidin production is also induced by inflammation [52]. In contrast, the body’s adaptation to iron deficiency at the systemic level is through the suppression of hepcidin [38], which increases FPN1, enhancing iron absorption in the gastrointestinal tract and the release of iron into the plasma by enterocytes and recycling macrophages [53]. Low levels of hepcidin promote the supply of iron to the bone marrow required for hemoglobin synthesis and red blood cell production [51].

The iron released by FPN1 is oxidized from Fe^2+^ to Fe^3+^ and then bound by transferrin, the main iron transport protein in plasma, responsible for the distribution of approximately 80% of this element in the body [44]. The TF molecule contains two globular domains, N and C, which allow the reversible binding of two Fe^3+^ ions [54]. Reduced plasma TF concentrations indicate a high saturation level of iron-binding sites, which may be indicative of iron overload [55]. In iron-deficient states, there is a compensatory increase in transferrin concentration with a concomitant decrease in transferrin saturation. Thus, transferrin acts as a biochemical marker of iron metabolism in the body [55].

LF, whose involvement in iron absorption processes in the gastrointestinal tract has been well documented, also plays an important role in iron metabolism. The mechanism involves the receptor-mediated absorption of lactoferrin-bound iron by small intestinal epithelial cells [56].

### 3.2. Iron Turnover at the Cellular Level

Cellular iron uptake depends on transferrin receptor 1 (TfR1), which recognizes iron-bound transferrin in peripheral tissues. TfR1 is expressed ubiquitously on the cell surface, and then, via the receptor, circulating iron is taken up by endocytosis [46,57]. After internalization, endocytic vesicles in the cell become acidified and iron is separated from transferrin and released into the cytoplasm via divalent metal transporter 1 (DMT1), after the prior reduction of Fe^3+^ to Fe^2+^ by STEAP3 [58]. TfR1 is recycled back to the cell surface. Transferrin is also released into the bloodstream and is available for further iron transport [46]. Iron transport by FPN1 is assisted by ceruloplasmin or its counterpart hephaestin, which oxidizes Fe^2+^ to Fe^3+^ before iron binds to Tf [32,46].

The regulation of the expression of proteins involved in iron metabolism and homeostasis (ferritin or transferrin receptors) is coordinated by the cooperative iron-detecting proteins IRP and iron-binding proteins IRE [58]. In iron-deficient cells, IREs bind to IRP 1 or IRP 2 and stabilize the mRNA, enhancing protein translation, increasing iron uptake, and inhibiting ferritin and ferroportin expression. This results in reduced unnecessary iron binding by ferritin and iron export by ferroportin, leading to increased levels of free iron available for cellular use. With excess iron in the cell, IRPs dissociate from IREs, which unlocks ferritin and ferroportin translation and reduces the stability of TfR and DMT1 mRNAs [58].

## 4. Anemia and Iron Overload in Oncology Patients

Anemia is a significant factor that worsens the course of the disease, quality of life, and prognosis of oncology patients [59]. It occurs in 20–40% of newly diagnosed patients and in as many as 80–90% of advanced-stage patients [60,61]. A specific form is cancer-related anemia (CRA), resulting from chronic inflammation, oxidative stress, metabolic abnormalities, and malnutrition, further intensified by treatment [14,62]. It is characterized by normocytic anemia (Hb 8–10 g/dL), low circulating iron levels (TSAT < 20%), and transferrin saturation occurring despite high iron stores [63,64]. Pro-inflammatory cytokines (TNF-α, IL-1β, IL-6, IFN-γ) play a key role in the pathogenesis, enhancing erythrocyte phagocytosis, inhibiting erythropoiesis, reducing iron availability and erythropoietin (EPO) synthesis, and increasing proptosis [62,65]. Chronic inflammation and oxidative stress induce hepcidin synthesis, which increases iron storage in macrophages and inhibits iron absorption from the gut, reducing iron availability for erythropoiesis up to 44% of the normal [66,67].

Tumor and anticancer therapies, such as chemotherapy and radiotherapy, can further damage the bone marrow, shorten erythrocyte survival time and increase hemolysis and microangiopathy [14,62]. Some patients develop absolute iron deficiency anemia (AID), characterized by TSAT < 20% and ferritin < 100 ng/mL, often caused by hemorrhage, malabsorption, or insufficient iron intake [9,60,64]. Despite the various causes of anemia, in both CRA and AID, limited iron availability for erythropoiesis leads to the development of anemia and its clinical consequences [9,68].

Excessive iron accumulation also promotes tumors, especially in patients with hemochromatosis, thalassemia, or chronic liver disease. Hereditary haemochromatosis increases the risk of colorectal, breast, and hepatocellular carcinoma [69,70,71]. Iron overload can also result from uncontrolled supplementation therapy [9].

## 5. Lactoferrin

### 5.1. Characteristics of Lactoferrin

Lactoferrin (lactotransferrin) is a highly glycosylated protein with a single polypeptide chain composed of 692 amino acids forming two globular lobes, linked by α-helix. LF was first isolated from cow’s milk in 1939 by Sorensen and Sorensen [72] and was identified in human milk in 1960 [73]. In 1993, Iyer and Lonnerdal identified LF in the secretions of exocrine glands and in neutrophil granules [74]. LF is a member of the transferrin family, with a similar amino acid composition (60% amino acid sequence identity), secondary and tertiary structure to serum TF, but different biological functions [75]. LF exists in three isoforms, of which only one, αLF, binds iron, while the other two, βLF and γLF, do not bind iron but express strong ribonuclease activity [76].

Human lactoferrin hLF can exist in many variants, the most important being the secreted full-length form (80 kDa) present in all secretions and body fluids of the human body (Table 1) [19,77] as well as in neutrophilic leukocyte granules [78] and the truncated intracellular form ΔLF (73 kDa), lacking much of the N-terminal region [79]. ΔLF is absent in other species, in contrast to the full-length form [80].

Under physiological conditions, hLF is a leading player in the regulation of iron absorption and metabolism. It is transferred via various receptors to and between cells, bile, serum, and cerebrospinal fluid [23]. hLF is likely to act as a negative regulator by suppressing the acute response to estrogen or infection through a sharp increase in protein expression [81]. Therefore, at the sites of infection and inflammation, the amount of secreted neurophilic hLF increases several-fold [19,23].

**Table 1:** Lactoferrin concentration in different human fluids and secretions [19,82,83,84,85].

Biological Fluids	Concentration	References
Colostrum	6–8 g/L	[19,82,83]
Human milk	1–4 g/L	[84]
Seminal plasma	>400–1900 µg/mL	[83,85]
Gastric	500–1000 µg/mL	[85]
Pancreatic	500 µg/mL	[85]
Nasal	100 µg/mL	[85]
Synoviral fluid	>10–80 μg/mL	[83,85]
Hepatic bile	10–40 μg/mL	[85]
Saliva	7–10 μg/mL	[83,85]
Vaginal secretion	8 µg/mL	[19]
Urine	1 ug/mL	[85]
Joint fluid	1 µg/mL	[19]
Blood	0.1–2.5 μg/mL	[85]

LF occurs naturally in mammalian milk and milk products. In human milk, hLF is the second most abundant whey protein with concentrations ranging from 1 to 4 g/L (6–8 g/L in colostrum), in cow’s milk from 0.3 to more than 4.0 g/L, in camel’s milk at 2.3 g/L, and in goat’s milk at 17.5 mg/L [82,86].

Currently, both recombinant hLF and bovine lactoferrin (bLF), isolated using dairy technology, are commercially available and can be added to food and clinical products [87]. Their chemical composition and functional properties show very high similarity [88]. LF obtained at an industrial scale is also derived from fungal, bacterial, and viral transgenic expression systems [23,89].

### 5.2. Properties of Lactoferrin

Among the many biological properties of lactoferrin, its ability to strongly bind and transport iron ions over a wide range of pH values is a key function, determining its effectiveness in regulating iron homeostasis [90]. Like TF, it shows the ability to bind Fe^3+^ with high affinity (KD~10^−20^ M) [91], but reversibly. However, unlike TF and other iron-binding proteins, LF can retain iron to a much lower pH (pH~3–4) occurring in response to existing inflammation in the body [92]. The ability of LF to bind iron bound in secretions and transferrin-bound iron in cells and circulation results in free iron of no more than 10–18 M [19]. LF can be both a supplier and a recipient of iron to cells. LF can act as an iron scavenger by both the direct iron chelation and modulation of major iron-related proteins [93]. LF is also capable of binding other metals to positively charged cations, including, among others, Al^3+^, Cr^3+^, Mn^3+^, Cu^2+^, Zn^2+^, Ni^2+^ [94], and various biological molecules such as DNA, heparin, or lipopolysaccharides (LPS) [91].

The ability of LF to bind iron is related to its structure. It consists of two homologous globular lobes (N-lobe and C-lobe), divided into two domains (N1 and N2) and (C1 and C2). The two N-lobes and the C-lobe are connected by a flexible, short bridge. The deep grooves between them can accommodate two Fe^3+^ iron ions [19,95,96]. The C-lobe in LF has a higher iron-binding capacity than the N-lobe [97]. The iron-binding sites in LF are more or less the same, regardless of the type of LF origin [98].

**Figure 1:**
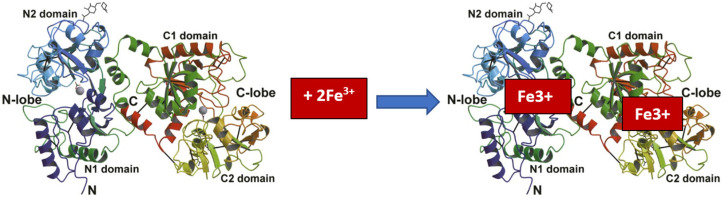
Fe^3+^ binding by lactoferrin [19].

Iron binding is reversible, so proteins can exist in iron-free (apo-LF) and iron-bound (holo-LF) forms (Figure 1) but also partially iron-bound (mono-LF) forms [99]. At the same time, it is possible for iron to be bound by one or both lobes of the LF molecule. Under physiological conditions in LF, only part of the iron-binding sites in the molecules are filled (usually 10–20%) [100].

LF’s ability to bind a variety of compounds and its molecular mechanisms affecting key chemical components of the host organism give it a wide range of biological activities beyond iron sequestration.

### 5.3. Efficacy of LF in Cancer Linked to Iron Chelation Capacity

Cancer cells are dependent on iron—it is essential for the initiation, growth, formation of the tumor microenvironment, and metastasis [101]. Therefore, they trigger mechanisms that increase iron influx and decrease iron efflux [9]. In addition, the frequent iron treatment of anemia in oncology patients may promote tumor cell survival by stimulating tumor growth and cell migration [102]. Although some studies indicate that increasing iron stores, both within cells and systemically, may paradoxically limit tumor progression [14].

The adaptation of tumor cells to increased iron requirements occurs through the overexpression of iron handling-related proteins such as transferrin receptor 1 (TFR1), metal transporter DMT1, and neutrophil gelatinase-associated lipocalin (NGAL). This process also includes the effects on ferritin, the dysregulation of IRP regulatory proteins, and the hepcidin-ferroportin pathway [14,42]. In addition, tumor cells can affect iron-dependent enzymes, including mitochondrial dehydrogenases and Krebs cycle enzymes [103].

LF, through its ability to chelate iron, can inhibit the activity of iron-containing proteins. This action is due to its ability to remove or sequester iron bound to transferrin, ferritin, and ‘transit’ intracellular iron [23]. LF interacts with the transferrin receptor 1 (TfR1) [104], increases ferroportin expression [105], and affects IRP regulatory proteins [106]. It also modulates other proteins associated with iron metabolism, such as membrane ceruloplasmin (Cp) and cytosolic ferritin (Ftn) [107], which may favor the restoration of iron homeostasis. In addition, LF shows a unique ability to penetrate into the cell nucleus, where it interacts with specific and non-specific DNA ligands [108,109].

By reducing iron availability in cancer cells, LF reduces ROS production and interferes with cellular functions that promote tumor growth. Decreased iron availability leads to a decrease in the activity of iron-dependent enzymes, which play a key role in the migration and invasion processes of cancer cells [110]. In addition, LF can induce ferroptosis, an iron-dependent form of cell death [111].

Cancer cells have the ability to evade death-inducing signaling. Meanwhile, LF has been described as a potent proapoptotic factor [112], which may be due to its ability to chelate iron, similar to that of pharmacological chelators such as desferrioxamine and deferiprone [113]. LF has a multilevel effect on iron metabolism—disrupting its homeostasis in cancer cells, leading to their dysfunction, while at the same time restoring the balance of this element in the body.

One postulated mechanism of action of LF, through which it activates signalling pathways leading to deleterious effects in cancer cells, may be its interaction with proteoglycans, glycosaminoglycans, and sialic acid, high levels of which are found in these cells [77].

Lactoferrin administration may also suppress pro-inflammatory activity. In addition, it promotes the transition of macrophages from a pro-inflammatory M1 phenotype to an anti-inflammatory M2 phenotype, leading to the increased production of anti-inflammatory cytokines, increased expression of Fpn, and restoration of natural iron export from macrophages into the bloodstream [114].

### 5.4. Anti-Inflammatory Properties of Lactoferrin

The presence of inflammation leads to the production of numerous immune mediators, such as cytokines and chemokines. These stimulate innate immune cells (including macrophages, dendritic cells, and neutrophils), which initiate the production of secondary mediators and the activation of the adaptive immune response [115]. Inflammation can both initiate cancer and contribute to its development and progression [116].

Human lactoferrin acts as an ‘alarmin’, released by immune cells in response to pathological conditions, activating both innate and adaptive immune responses [117,118]. Bovine lactoferrin (bLF) shows high sequence homology with hLF and has similar anti-inflammatory properties, although it may act through different mechanisms [18,19].

A key role in the anti-inflammatory effect of hLF is played by its ability to sequester iron and regulate the levels of reactive oxygen species (ROS), thereby limiting the damage caused by excessive inflammatory responses. hLF binds two Fe(III) ions per molecule, even at the low pH characteristic of the inflamed and infected sites [119]. LF influences the immune response by modulating T and B cell activity [120]. It reduces the production of pro-inflammatory cytokines and stimulates the synthesis of anti-inflammatory cytokines [121]. It inhibits changes in the proteins involved in iron homeostasis in inflamed macrophages and prevents excessive intracellular iron storage. Its action includes both the prevention and alleviation of already existing inflammation. It reduces the levels of the cytokines IL-6 and IL-1β, while restoring the levels of ferroportin, ceruloplasmin (Cp-GPI), ferritin, and transferrin receptor 1 (TfR1) to the physiological values. This allows iron to be efficiently exported from cells into the bloodstream, preventing iron accumulation and iron deficiency (IDA) and chronic disease anemia (ACD) [107]. Different variants of hLF (rtHLF4, rteHLF1, and rpHLF2) showed anti-inflammatory effects in human cell lines [122]. The anti-inflammatory effects of hLF also include effects on the NF-κB pathway, a key regulator of inflammatory processes [18].

It is worth noting, however, that in some studies, LF showed conflicting effects in different in vitro models, most likely due to the different experimental conditions [19].

### 5.5. Bacteriostatic and Antiviral Effects of Lactoferrin

LF exhibits broad antibacterial, antifungal, and antiparasitic activities [117,118,123]. Chronic bacterial infections are recognized as an important risk factor in the development of some cancers [124]. Long-term inflammation caused by chronic infections can lead to DNA damage and cause cell mutations, which can in turn promote cancer development [125]. LF binds iron, limiting its availability to pathogenic bacteria, which inhibits their proliferation. It has also been shown that iron-binding sites in lactoferrin can interact with bacterial cell walls, disrupting their integrity and leading to cell death. Additionally, it increases the susceptibility of bacteria to antimicrobial agents. Its antimicrobial activity against many strains has been confirmed [118], but the effectiveness of LF in inhibiting bacterial growth depends on the dose, the type of the strain, and the specific product [126]. The antiviral activity of LF has been documented against several viruses, including Zika, dengue, hepatitis, and influenza [127]. It has been shown that LF can also prevent interactions between viruses and certain cellular receptors, including SARS-CoV-2 [18,23]. Additionally, through other mechanisms of action, it can prevent SARS-CoV-2 coronavirus from attaching to host cells [23].

### 5.6. Lactoferrin Versus Iron-Driven ROS

The production of certain amounts of nitrogen-oxygen free radicals (ROS/RNS) is essential for the normal functioning of the body [128,129]. Under physiological conditions, their excess is removed from the body through antioxidant processes. However, under pathological conditions, an imbalance between the production and neutralization of ROS can lead to oxidative stress, lipid peroxidation, DNA and protein damage, and the activation of inflammatory processes [4,24,130].

#### 5.6.1. Iron and Its Role in ROS Generation

**Figure 2:**
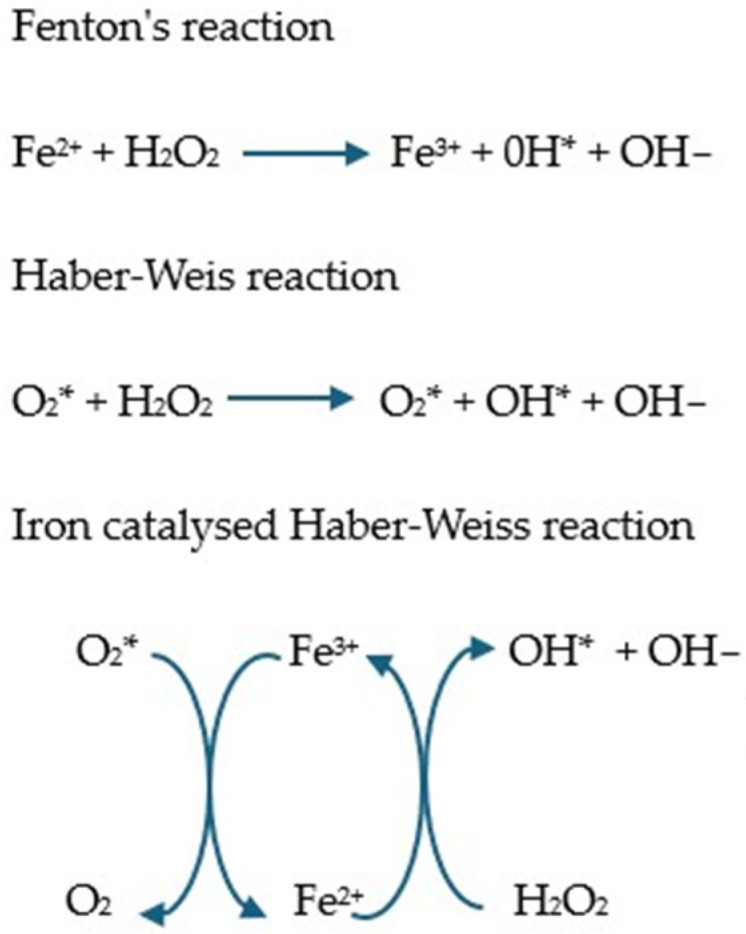
Fenton and Haber–Weiss reaction and iron catalyzed by the Haber–Weiss reaction [133,134,135,136]. In the Fenton reaction, Fe^2+^ reacts with hydrogen peroxide and is oxidized to Fe^3+^ with the formation of hydroxyl radicals. In the presence of superoxide, the Fe^3+^ produced in the Fenton reaction can be reduced to Fe^2+^, after which the Fenton reaction proceeds anew, which is called the Haber–Weiss reaction [5].

Iron, due to its ability to exist on two oxidation levels (Fe^2+^ and Fe^3+^), has a high capacity to generate ROS [131]. Under physiological conditions, its metabolism is controlled to prevent the excessive accumulation of the free form. However, in pathology, highly reactive unbound iron can be formed, generating oxidative stress in the Fenton and Haber–Weiss reactions (Figure 2) [132]. In particular, the hydroxyl radical is a potent oxidant causing lipid peroxidation and apoptosis [128].

In cancer, excess free iron (intracellular, labile iron pool LIP; and extracellular, labile plasma iron LPI) contributes to the formation of ROS [137]. Under physiological conditions, unused iron is either used as an enzyme cofactor or stored in liver cells [38,40]. In pathology, an excessive increase in LIP can overwhelm the cells’ antioxidant capacity [138].

Exceeding the iron-binding capacity of transferrin results in the formation of non-transferrin-bound iron (NTBI), which can penetrate cells and lead to iron overload [139]. Stable plasma iron (LPI), a component of NTBI, can penetrate cells via unregulated pathways and increase LIP levels, leading to clinically relevant iron overload [140]. Similarly, increased ROS production may result from blood transfusion therapy [141].

Interestingly, while moderate increases in ROS promote tumor cell proliferation and survival [129], high levels of ROS can induce ferroptosis—iron-dependent, non-apoptotic cell death—and drive oxidative stress-induced tumor clearance [8,9]. ROS-related cancer treatment models rely on both reducing and increasing ROS levels.

#### 5.6.2. Antioxidant Properties of Lactoferrin

LF, due to its ability to chelate iron, reduces its availability for the Fenton reaction, which reduces oxidative stress [118]. It also promotes antioxidant defense mechanisms and possibly directly scavenges free radicals [142]. The antioxidant effects of LF have been demonstrated in numerous in vitro (Table 2) and human studies (Table 3).

In cancer and non-cancer cell lines, LF of various origins in different forms not only reduced ROS production [143,144,145,146] and significantly increased ferric reducing antioxidant capacity (FRAP) [147], but they also decreased the activity of antioxidant enzymes such as catalase, glutathione peroxidase (GPx), glutathione reductase (GR), glutathione-S-transferase (GsT), and superoxide dismutase (SOD) [145,148]. LF also modulated the transcription of antioxidant-related genes and regulated the iron transport system, reducing lipid peroxidation and DNA damage in astrocytes [144,149]. In addition, it inhibited hydrogen peroxide-induced apoptosis by inhibiting caspase-3 and Akt activation [143].

LF also reduced the levels of hydroperoxides, whose breakdown leads to the formation of toxic reactive compounds that damage tissues [147]. Studies have demonstrated its ability to inhibit TNF-α-induced ROS production in non-malignant colorectal fibroblasts and in cancer cells, to inhibit the expression of pro-inflammatory factors [122].

In animal models, LF showed the ability to reverse oxidative stress in the brain and activated signaling pathways, promoting the expression of genes involved in antioxidant defense [150]. In addition, its ability to interact with specific cellular receptors (LfRs) has been documented to induce the activation of signalling pathways promoting the expression of genes involved in antioxidant defense [150]. These properties allow LF to play a key role in regulating oxidative stress and inflammatory processes, making it a potential therapeutic tool in many chronic diseases and cancers.

Human studies have confirmed that the antioxidant properties of LF supplementation resulted in an increase in hydrophilic antioxidant capacity [151], a decrease in oxidative stress markers [152,153], and an increase in total antioxidant status (TAS) [154]. It also improved the levels of antioxidant markers, such as SOD, GPx, and glutathione, compared with the placebo group [153].

Supplementation with LF-containing colostrum led to lower levels of thiobarbituric acid reactive substances (TBARS). Higher levels of haemopexin, an antioxidant that binds free heme, were observed in trained female athletes both immediately after exercise and after 3 h of restitution [155]. In addition, in patients with Alzheimer’s disease, LF supplementation led to the decreased expression of phosphatase and tensin homolog (PTEN), tau, and mitogen-activated protein kinase (MAPK1), as well as decreased serum levels of Aβ42, which may indicate its beneficial effects on oxidative stress and inflammatory processes [152].

Another proposed antioxidant mechanism of LF is its ability to counteract the so-called oxygen explosion in neutrophils, which results in the production of cell-damaging free radicals [77]. LF may also act on the cell nucleus to facilitate the translocation of Nrf2, a key regulator of the antioxidant response [114].

**Table 2:** Antioxidant effects of lactoferrin in selected in vitro studies.

Tests	Cell Lines	Lactoferrin Used in the Test	Results
Safaeian et al. [147]	Human umbilical vein endothelial cells (HUVECs) subjected to H_2_O_2_	Sigma-Aldrich human LF (St. Louis, MO, USA) at concentrations of 6.25–100 μg/mL	- LF at concentrations of 6.25–100 μg/mL significantly increased FRAP levels in intracellular fluid and at concentrations of 12.5–100 μg/mL in extracellular fluid. - LF at concentrations of 6.25–100 μg/mL significantly decreased intracellular and extracellular hydroperoxide levels compared to the control group.
Hou et al. [144]	SH-SY5Y cells from the SK-N-SH neuroblastoma tumor cell line (cell model for neurodegenerative disorders)	bird’s nest (EBN) and its components, lactoferrin (LF) and ovotransferrin (OVF), induced by H_2_O_2_	- EBN and its components attenuated H_2_O_2_-induced cytotoxicity and reduced radical oxygen species (ROS) through increased scavenging activity. - LF, OVF, and EBN induced transcriptional changes in antioxidant-related genes that tended to be neuroprotective compared to the H_2_O_2_-treated group
Burrow et al. [145]	HT29 colon cancer cells exposed to H_2_O_2_	Apo-bLF and bLF 100% iron-saturated (Fe-bLF)	- Significant reduction in the activity of antioxidant enzymes (catalase, glutathione peroxidase (GPx), glutathione reductase (GR), glutathione-S-transferase (GsT), and superoxide dismutase (SOD)) after treatment with Apo-bLF or Fe-bLF, with or without exposure to H_2_O_2._
Pan et al. [122]	Human non-neoplastic colorectal fibroblasts CCD-841-CON and CCD-18co, - human colorectal adenocarcinoma cells HT29	rekombinacyjnie wyrażone fragmenty laktoferryny rtHLF4, rteHLF1 i rpHLF2	In non-malignant cells, flHLF and lactoferrin variants (rtHLF4, rteHLF1, and rpHLF2) inhibited TNF-α-induced ROS generation. In cancer cells, rteHLF1 and rpHLF2 had no effect on ROS production. rtHLF4 in both TNF-α-treated fibroblast cells resulted in a decrease of more than 40% in ROS produced after treatment with 10 μM protein compared to untreated CCD-841-CON and CCD-18co fibroblast cells, and rteHLF1 and rpHLF2 reduced ROS generation by 10–20%. rtHLF4 also showed the highest suppression of ROS generation in HT29 cells after treatment with 0.1 μM protein. flHLF and rpHLF2 could suppress ROS generation to a lesser extent, while rteHLF1 showed no inhibition of ROS generation.
Ianiro et al. [149]	Human stellate glioma cells (U373-MG) and human neuroblastoma cells (SH-SY5Y) constitutively expressing the HIV-1 Tat viral protein (U373-Tat).	BLF native (Nat-bLF) Iron saturation~11%, and iron saturation (Holo-bLF) > 95%. (100 μg/mL)	In human cells (U373-Tat), both Nat-bLF and Holo-bLF increased the host antioxidant response by up-regulating System X c—and the cellular iron exporter Ferroportin via the erythroid nuclear factor 2 (Nrf2) pathway, thereby reducing ROS-dependent lipid peroxidation and DNA damage in astrocytes.
Park et al. [143]	Human mesenchymal stem cells (hMSCs) exposed to H_2_O_2_	Lf Sigma (USA)	- Inhibition of intracellular ROS production induced by hydrogen peroxide. - Reducing hydrogen peroxide-induced apoptosis through the inhibition of caspase-3 and Akt activation.
Burrow et al. [148]	HT29 human colorectal epithelial cancer cell line exposed to H_2_O_2_	98% selenium-saturated form of bLF	- Changes in the activity of all antioxidant enzymes (glutathione peroxidase (GPx), glutathione reductase (GR), glutathione-s-transferase (GsT), catalase and superoxide dismutase (SOD)).
Zhang et al. [146]	RAW264.7 macrophages stimulated with lipopolysaccharide	LF and Cu-enriched LF products: 1. 0.16 Cu mg/g LF, 2. 0.32 Cu mg/g LF, doses of 10–80 μg/mL	- LF and Cu-enriched products at doses of 10 and 20 μg/mL showed different effects on stimulated cells, by partially reducing or increasing ROS production depending on the Cu enrichment and dose levels used. - Compared to LF, Cu-enriched LF (0.16 mg Cu/g LF) at 10 μg/mL showed increased inhibition of ROS production, and the inhibition of the Cu-enriched LF product (0.32 mg Cu/g LF) at 20 μg/mL on ROS production was reduced.

LF—lactoferrin; bLF—bovine lactoferrin; Apo-bLFA—iron-free bLF molecule; FRAP—ferric reducing antioxidant power; H_2_O_2_—hydrogen peroxide; Cu—copper.

**Table 3:** Antioxidant properties of LF in human studies.

Tests	Type of Study	Dose/Intervention Time	Test Group	Results
Mulder et al. 2008 [151]	Dose-response study	1 placebo capsule for 7 days, 100 mg bovine lactoferrin (bLF) (Glycomax Lactoferrin) for 7 days, followed by 200 mg lactoferrin. Intervention time: 7 days	8 healthy men aged 30 to 55 years	Statistically significant increases between pre-supplementation and post-200 mg supplementation levels in the hydrophilicity of the antioxidant capacity.
Cieślicka et al. 2022 [155]	Clinical trial	Bovine colostrum supplements (total 3.2 g; in divided doses 4 times daily) produced by AGRAPAK, Poland. Intervention time: 6 months	20 highly trained female athletes (11 in the colostrum supplementation group/9 in the placebo group)	Compared with the placebo group, the colostrum group showed a significant decrease in thiobarbituric acid reactive substance (TBARS) levels at all time points, while a marked increase was observed for superoxide dismutase (SOD) activity. In the colostrum-supplemented group, higher hemopexin levels were observed immediately after exercise, as well as after 3 h of restitution.
Derosa et al. 2020 [153]	Randomized trial	Undenatured whey protein isolate (WPI; ≥92.5%) with high native cysteine content (2.7%) and standardized lactoferrin content (≥0.7%) Intervention time: 3 months.	120 white patients with type 2 diabetes and glycosylated hemoglobin ≥6.5%. Patients received daily supplementation with (1) WPI or (2) placebo.	Markers of oxidation (SOD, glutathione peroxidase, glutathione, and the ratio of reduced glutathione to oxidized glutathione) were significantly lower in the WPI group than in the placebo group
Mohamed et al. 2019 [152]	Randomized trial	Group 1: Alzheimer’s patients without LF. Group 2: LF capsules (Jarrow Formulas^®®^, USA, 250 mg/day, LF).	50 patients with a clinical diagnosis of probable Alzheimer’s disease (28 men and 22 women) Intervention time: 3 months	Serum antioxidant markers such as MDA, glutathione, total antioxidant capacity (TAC), and nitric oxide (NO) improved significantly after the daily administration of LF. There was a significant decrease in the expression of the PTEN, tau, and MAPK1 genes and serum amyloid A42.
Trentini et al. 2020 [154]	Randomized trial	Group 1: (*n* = 20) vaginal lactoferrin 300 mg 4 h before amniocentesis, Group 2: (*n* = 20) intravaginal lactoferrin 12 h before amniocentesis, Group 3: no treatment	60 pregnant patients undergoing amniocentesis at week 16 randomized in a 1:1:1 ratio to 3 groups	Administration of lactoferrin 4 h before running decreased thiobarbituric acid reactive substances (TBARSs, as a measure of ROS) and the oxidative stress index (OSI), and it increased the total antioxidant status (TAS). Administration of lactoferrin 12 h earlier was associated with a decrease in TBARSs, but to a lesser extent. There was no statistically significant difference for OSI or TAS.

LF—lactoferrin.

### 5.7. Lactoferrin and the Microbiota

The gut microbiota plays a key role in the regulation of health, and its disruption can lead to inflammatory and cancerous diseases of the gastrointestinal tract. Carcinogenesis in the gut is driven by the presence of microorganisms, the existence of inflammation, and the modulation of intestinal immunity [156]. The relationship between pathology and the structure and function of the gut microbiome is bidirectional—the gut microbiota influences the inflammatory microenvironment, and inflammation can alter its composition [157]. Oxidative stress, autophagy, and fatty acid metabolism also play a role in tumorigenesis. Colorectal neoplasms, in particular, have been associated with dramatic changes in gut microbial composition [158]. Colorectal cancers have an inflammatory basis [156].

Iron can influence cancer biology by altering the microbiome [14]. Both its excess and deficiency affect dysbiosis [16]. Iron supplementation in infants decreased *Bifidobacteria* and increased the number of *Enterobacteriaceae* and markers of intestinal inflammation [159]. Iron administration also led to a decrease in the relative abundance of lactic acid bacilli and potentially increased susceptibility to bacterial infections [160].

Iron deficiency may promote intestinal infections, including by reducing the number of lactic acid bacteria [161]. It may also contribute to the development and progression of colorectal cancer, leading to genomic instability, resistance to therapy, and an impaired immune response.

LF shows a beneficial effect on the composition of the microbiota, promoting the growth of beneficial bacteria such as *Bifidobacterium* and *Lactobacillus*, while inhibiting the growth of pathogens such as *Escherichia coli*, *Salmonella*, and *Shigella*. This action is mainly due to its ability to chelate iron, which is essential for the growth of many pathogenic bacteria. Depriving these bacteria of the free iron necessary for growth leads to their death [162,163]. Its action is due to the chelation of iron, which inhibits the growth of pathogenic bacteria. Thus, it promotes the balance of the intestinal microbiota. LF has a bactericidal effect against Gram-positive and Gram-negative microorganisms and inhibits the action of siderophores—organic chemicals that chelate iron ions, secreted by some bacteria to capture this element and transport it into the cell [23]. Its antibacterial activity does not adversely affect most probiotic strains, allowing the selective modulation of the intestinal microbiota [164]. It can also interfere with bacterial osmosis, leading to the elimination of bacteria [165]. Studies have shown that LF supplementation improves intestinal maturation and reduces pathogens in piglets [166]. In addition, LF exhibits antiviral and antifungal properties, which can promote the balance of the intestinal microbiome. It inhibits the growth of viruses and their ability to invade [162]. A meta-analysis suggests that it may reduce the risk of fungal sepsis and shorten the hospitalization of preterm infants [167]

## 6. Inhibition of Anemia by Lactoferrin

LF administration is a promising therapy for iron deficiency (ID) conditions, including iron deficiency anemia (IDA), both as a primary treatment and as an adjunctive therapy. Due to its ability to inhibit interleukin-6 (IL-6) expression, LF decreases hepcidin synthesis, which in turn increases ferroportin levels, restoring iron export from cells to the blood in a hepcidin-dependent or -independent manner and is thus also effective in the treatment of CRA [168]. In addition, LF induces an increase in transferrin receptor 1 (TfR1) levels and a decrease in ferritin (Ftn) levels [19].

Numerous intervention studies have confirmed the efficacy of LF supplementation, showing an increase in hemoglobin (Hb), total iron, erythrocyte count (RBC), and serum ferritin levels. In addition, improvements in erythropoiesis indices such as mean red cell volume (MCV) and mean erythrocyte hemoglobin content (MCH) were noted. At the same time, an increase in transferrin saturation and a decrease in hepcidin and the total iron binding capacity (TIBC) were observed compared to baseline values [168,169,170,171,172,173,174,175,176]. Importantly, a concomitant decrease in IL-6 was observed in many cases [168,172,173,174,175]. A recent systematic review and meta-analysis confirmed the efficacy of bLF at doses of 100–250 ng/day in patients with low Hb levels [177].

Interestingly, the administration of iron-saturated bLF at only 20–30% (70–84 µg/day of iron vs. 1–2 mg of the required daily dose) already had a significant effect. While the use of bLF in women with iron deficiency (ID) or iron deficiency anemia (IDA) decreased IL-6 levels and increased prohepcidin levels, in patients without ID/IDA, bLF did not affect IL-6 levels but increased prohepcidin levels [178], indicating a selective effect dependent on body iron status.

Most studies have shown similar or superior efficacy of bLF to traditionally used therapy (ferrous sulfate and ferric hydroxide) in restoring iron deficiency, with significantly fewer gastrointestinal side effects [168,170,171,172,173,174,179]. bLF may be a beneficial alternative in the treatment of inflammation-associated anemia, especially in patients who experience the side effects of traditional iron therapies. It may also be a valuable option in oncology patients with anemia, in whom iron supplementation may lead to the increased secretion of pro-inflammatory IL-6. The collected results of the study are shown in Table 4.

## 7. Efficacy of Lactoferrin from Dietary Sources and New Directions

LF, which is found in cow’s milk, cow milk products and milk mixtures, and is used in the production of ice cream, biscuits, and processed cereal products [77], appears to be an ideal candidate with potential health benefits in the prevention and treatment of cancer, as well as in the reversal of the adverse effects of therapies. It can be easily introduced into the habitual diet due to the palatability of its nutritional carriers, its high availability, and low price. And oral administration is the most convenient and safest form of administering an ingredient to benefit the body. In addition, its high tolerability and safe profile (European Food Safety Authority) [180], excluding people with cow milk protein allergy [77], speak in its favor.

However, although in clinical trials LF preparations taken in average doses of 100–200 mg/day have shown high efficacy in the treatment of several diseases, the same effect has not been demonstrated for LF consumed with food products. On average, in mature cow’s milk, its content ranges from 0.1 to 0.3 mg/mL, which may be too low for preventive or therapeutic efficacy. In a study to evaluate the efficacy of bLF in the treatment of certain types of precancerous lesions to prevent their transformation into highly cancerous cells, only the ingestion of 3.0 g bLF significantly delayed the growth of adenomatous polyps, while lower doses had no significant effect [181]. Similarly, in infants with anemia, only higher doses resulted in a significant increase in Hb levels, whereas lower doses did not [169].

Orally administered LF has low bioavailability due to degradation at unfavorable gastric pH and by proteolytic enzymes in the intestinal lumen and poor permeability through the intestinal epithelium, which limits its effective delivery to target sites. LF is much better absorbed when administered directly into the duodenum [182].

Therefore, novel methods of LF delivery are being developed, including coating enteral capsules containing LF, encapsulating it in nanocarriers, which allows it to be released at a site with less gastric pepsin activity, increasing its bioavailability by reaching the small intestine intact [183,184]. Various nano- and microparticle carriers have been proposed for enhancing LF delivery in various therapies, including liposomes, polymers, and solid lipid nanoparticles modified with chitosan or pectin. Several carriers for intraluminal delivery are also being investigated [112,184]. In addition, alternative strategies to increase the bioavailability of LF, such as conjugation with folic acid or other ligands that stabilize the protein in the gastrointestinal environment, are being investigated. Other routes of administration, including transdermal or inhalation, are also being considered, which could avoid degradation in the gastrointestinal tract.

Several results from in vivo and in vitro studies indicate that LF delivered in this way counteracted oxidative stress and, in some cancers, inhibited colony growth, reduced tumor volume, and caused the complete regression of the tumor volume [185,186]. In addition, studies suggest that LF may act synergistically with other therapies such as chemotherapy, immunotherapy, and targeted therapy [112,187]. It may increase the sensitivity of cancer cells to cytostatic agents, improving treatment efficacy. Additionally, the use of probiotic bacteria as internal producers of LF in the body may open new perspectives for its therapeutic use.

The appropriate dose of LF and its dietary source that would make it effective in the prevention or treatment of cancer seem areas worthy of exploration in future research. This is a difficult problem to solve, especially as the concentration of LF in milk is variable, fluctuating within quite large limits and depending on the lactation period. However, the benefits that could arise from the introduction of LF as a component of the usual diet could resonate in reducing morbidity, facilitating treatment, and alleviating the severity of the cancer course.

## 8. Conclusions

Lactoferrin exhibits potent anticancer effects due to its ability to bind iron and regulate its metabolism. Iron is a key element for cancer cell proliferation, and excess iron can promote tumorigenesis by generating oxidative stress. By sequestering iron, LF limits its availability to cancer cells, inhibiting their growth, migration, and invasion. This mechanism involves several key processes, such as the modulation of oxidative stress, regulation of signaling pathways involved in iron metabolism, effects on apoptosis and ferroptosis, and modulation of the gut microbiota.

One of its main mechanisms of action is the reduction of oxidative stress, which promotes DNA mutations and cancer progression. LF blocks the Fenton reaction, reducing the production of reactive oxygen species (ROS), while stimulating the body’s antioxidant mechanisms.

By limiting iron availability, LF also affects tumor metabolism—it reduces ferritin and transferrin receptor 1 (TfR1) expression, which limits iron uptake by cancer cells, while increasing ferroportin levels, facilitating the removal of iron from cells.

LF also induces ferroptosis—iron-dependent cell death, which leads to excessive lipid oxidation in cell membranes and the destruction of cancer cells. In addition to this, it has an immunomodulatory effect by inhibiting the pro-inflammatory NF-κB pathway and reducing the production of cytokines, such as IL-6, which promote cancer progression. Additionally, LF influences gut microbiota, promoting the growth of beneficial bacteria and reducing the growth of iron-using pathogens, which may be important in the prevention of colorectal cancer.

LF also shows potential in the treatment of cancer anemia—by regulating levels of hepcidin and IL-6, it restores normal iron transport, minimizing the risk of both iron deficiency and iron over-accumulation, which can promote cancer development.

With its ability to regulate iron homeostasis and its multidirectional anti-tumor effects, LF represents a promising therapeutic agent that may find application in both the prevention and treatment of cancer.

Further clinical studies should focus on optimizing dosage, bioavailability, and potential synergy with other oncology treatments. It may also be possible to use LF in personalized therapies, taking into account the individual patient needs, tumor type, and gut microbiome profile. Its safety and low toxicity make it a valuable complement to conventional therapeutic strategies.

## Figures and Tables

**Table 4:** Review of studies on the efficacy and safety of LF in anemia.

Author	Test/Intervention Time	Dose	Study Group	Results
Lepanto et al. 2018 [168]	Study period: 30 days	Women included in Arm A, C, E, and G bLF oral 100 mg 20–30% iron-saturated (70–84 μg elemental iron) 2× daily, women included in Arm B, D, F, and H standard Italian therapy—oral 329.7 mg ferrous sulfate 1× daily (105 mg elemental iron).	- 20 pregnant and 9 not pregnant women, anemia and β-thalassaemia; 70 pregnant and 79 not pregnant with hereditary thrombophilia (HT) affected by anemia of inflammation (AI). - 20 pregnant women with anemia suffering from various pathologies.	In anemic pregnant and nonpregnant women with minor β-thalassemia, bLF decreased IL-6 and increased total serum iron (TSI) concentrations. bLF was more effective than ferrous sulfate in the treatment of AI in pregnant and nonpregnant women with HT, reducing both serum IL-6 and hepcidin, increasing hematological parameters such as red blood cell counts (RBCs), hemoglobin (Hb), TSI and serum ferritin. bLF has been effective in treating anemia in other pathological pregnancies.
Mahmoud et al. 2023 [175]	Study period: 6 months	1. 100 mg 20–30% iron-saturated bLF (70–84 μg iron) 2× daily 2. 576 mg iron glycine sulfate (100 mg iron) 2× daily	70 bLF/70 iron glycine sulfate patients on regular hemodialysis with iron deficiency anemia.	bLF significantly reduced serum hepcidin levels and significantly increased Hb concentration and transferrin saturation (TSAT), significantly more strongly than iron (II) glycine sulfate. Iron (II) glycine sulfate significantly decreased serum hepcidin levels and significantly increased Hb and TSAT.
El Amrousy et al. 2022 [174]	Study period: 3 months	1. Ferrous sulfate 6 mg/kg/day 2. LF 100 mg/day	80 children with inflammatory bowel disease (IBD) and iron deficiency anemia (IDA), iron sulfate group (n = 40), lactoferrin group (n = 40).	Hb, mean blood cell volume, serum iron, transferrin saturation, and serum ferritin significantly increased, while total iron binding capacity (TIBC) significantly decreased after ferrous sulfate or LF administration compared with the baseline data. LF significantly increased Hb, serum iron, TS, and serum ferritin compared to ferrous sulphate. LF significantly decreased IL-6 and hepcidin levels.
Paesano et al. 2010 [178]	Study period: 30–90 days	1. bLF 100 mg/day saturated with iron about 30% 2. Ferrous sulfate 520 mg/day 3. Control group	71 pregnant/189 non-pregnant women with iron deficiency and iron deficiency anemia Arm A: with bLF (30/90), Arm B: with ferrous sulfate (30/90), Arm C: control group (11/9).	In pregnant women, bLF decreased serum IL-6 levels and increased prohepcidin. In non-pregnant women, bLF did not alter low levels of IL-6, while it increased prohepcidin. Ferrous sulfate increased IL-6 levels and decreased prohepcidin.
Paesano et al. [173]	Study period: from inclusion to delivery	1. Orally 100 mg bLF 2× daily 2. 520 mg ferrous sulfate 1× daily	295 pregnant women with hereditary HT thrombophilia (≥18 years) suffering from anemia (156 women)/iron deficiency anemia (139 women).	Red blood cells, Hb, total serum iron, and serum ferritin were significantly higher in the bLF-treated group than in the iron sulfate group. Serum IL-6 levels decreased significantly in women treated with bLF and increased in women treated with iron sulfate, bLF had no adverse effects. Adverse effects were reported in 16.5% of women treated with ferrous sulfate.
Nappi et al. 2009 [176]	Study period: 30 days	1. 100 mg bLF 2× daily (group A; n = 49) 2. 520 mg ferrous sulfate 1× daily (group B; n = 48)	97 pregnant women with iron deficiency anemia, bLF (n = 49), ferrous sulfate (n = 48).	In both groups, Hb, serum ferritin, and iron were significantly increased, while TIBC was significantly decreased compared to baseline values. No significant differences were observed between groups. The median scores for abdominal pain and constipation were significantly higher in patients treated with ferrous sulfate compared to patients treated with bLF.
Rosa et al. 2020 [172]	Study period: 30 days	bLF (100 mg 2× daily) orally 1. Before or 2. during meals	Pregnant women with hereditary thrombophilia and inflammatory anemia.	A significant increase in RBCs, Hb, TSI, and serum ferritin levels, together with a significant decrease in interleukin-6, was detected in group A, but not in group B.
Chen et al. 2020 [169]	Intervention time: 3 months	Milk mixture enriched with bLF: group 1 with bLF concentration of 38 mg/100 g, group 2 with 76 mg/100 g bLF, and group 0 without bLF	108 infants born at term, aged 6–9 months.	Formula enriched with 76 mg/100 g bLF significantly increased infants’ Hb levels compared to infants in the other two groups
Paesano et al. 2006 [170]	Study period: 30 days	1. Oral1 administration of ferrous sulfate (520 mg once daily) 2. 30% iron-saturated bovine lactoferrin (bLF) (100 mg twice daily) 3. Control group without treatment	300 women in various trimesters of pregnancy.	In women without treatment, Hb and total serum iron values decreased significantly, especially in women 18–31 weeks of pregnancy. After 30 days of oral administration of bLF, Hb and total serum iron values increased and to a greater extent than observed in women treated with ferrous sulfate, irrespective of the trimester of pregnancy. In contrast to ferrous sulfate, bLF did not cause any side effects.
Omar et al. 2021 [171]	Intervention time: 4 weeks	1. Iron hydroxide-polymaltose complex IPC 6 mg/kg/day of elemental iron in 2 divided doses 2. LF (30% iron-saturated)	66 children aged 1–10 years with cerebral palsy and iron deficiency anemia, 1. 32 children randomly received IPC, 2. 34 received LF 200 mg per day.	In both the IPC and LF groups, significant improvements in Hb, ferritin and serum iron, total iron binding capacity, mean red cell volume, and mean red cell hemoglobin compared to baseline values were observed. Adjusted mean changes in Hb and SF in the LF group were significantly higher than in the IPC group. Constipation was less frequent in the LF group than in the IPC group.

## Data Availability

Not applicable.
